# Supplementary figures and images for: Fluctuations in auxin levels depend upon synchronicity of cell divisions in a one-dimensional model of auxin transport
Source: PLoS Comput Biol. 2023 Nov 30;19(11):e1011646. doi: 10.1371/journal.pcbi.1011646 (PMC10688697; doi:10.1371/journal.pcbi.1011646)

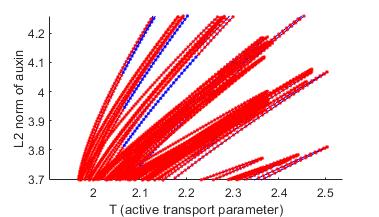

Supplement: S1 Fig — A refined and zoomed version of Fig 1 showing additional, unstable branches. (JPG) [file pcbi.1011646.s001.jpg]

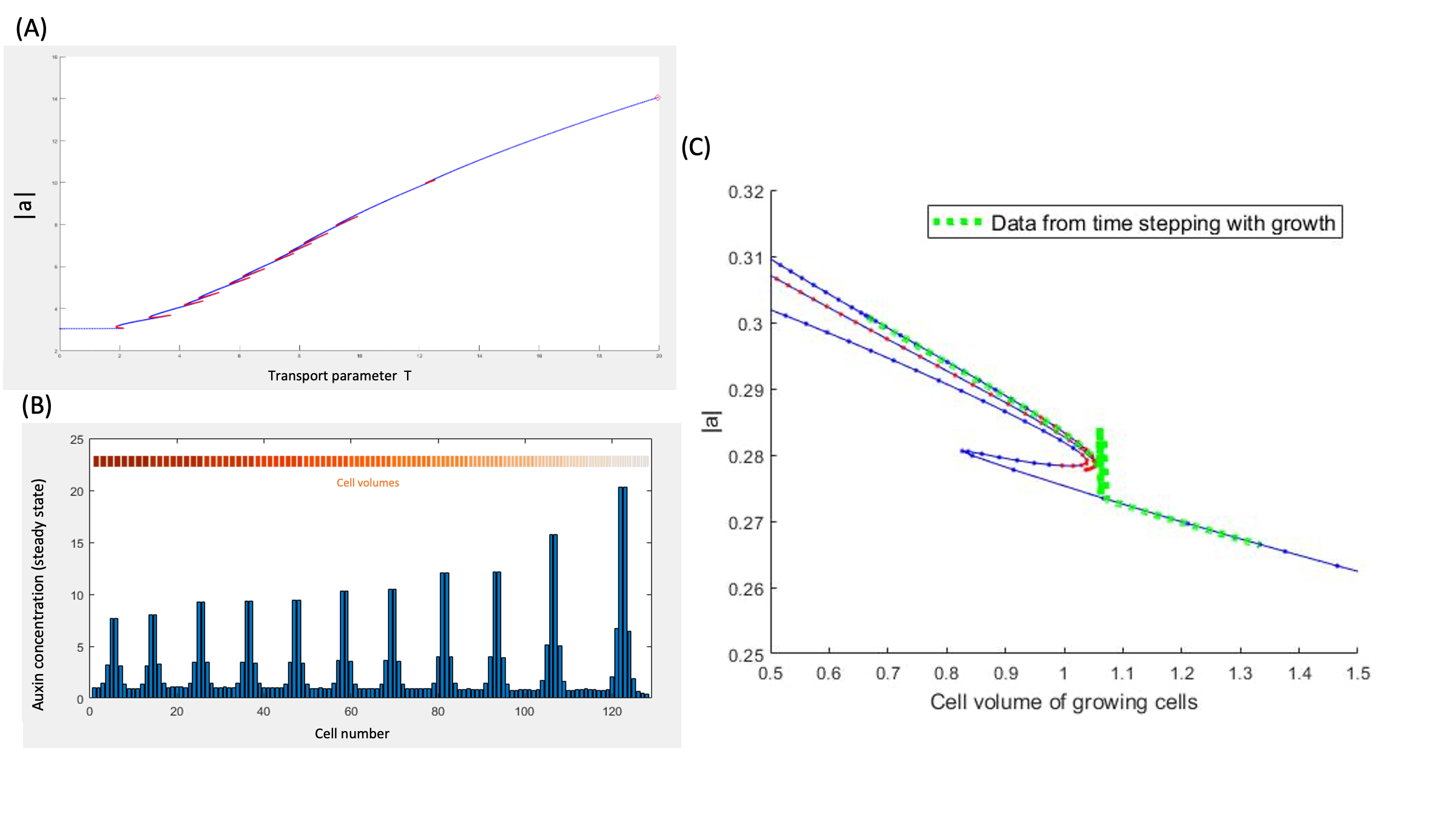

Supplement: S2 Fig — As a complement to Fig 2, alternative diagrams are included. (A) Continuation in T for a domain comprising 16 groups of 8 identical cells whose volume increase from 1 (8 tip cells) to 10 (leftmost 8 cells). (B) Steady state (auxin concentration) for T ≈ 20; the larger cells require higher transport rates to create multiple auxin maxima. Patterns for lower T have fewer auxin peaks. Cell sizes are represented in the insert. (C) Continuation using cell volume as control parameter can be used to illustrate the creation/annihilation of auxin peaks leading to an oscillatory signal. Axes labels are self-explanatory, T = 2. (PNG) [file pcbi.1011646.s002.png]

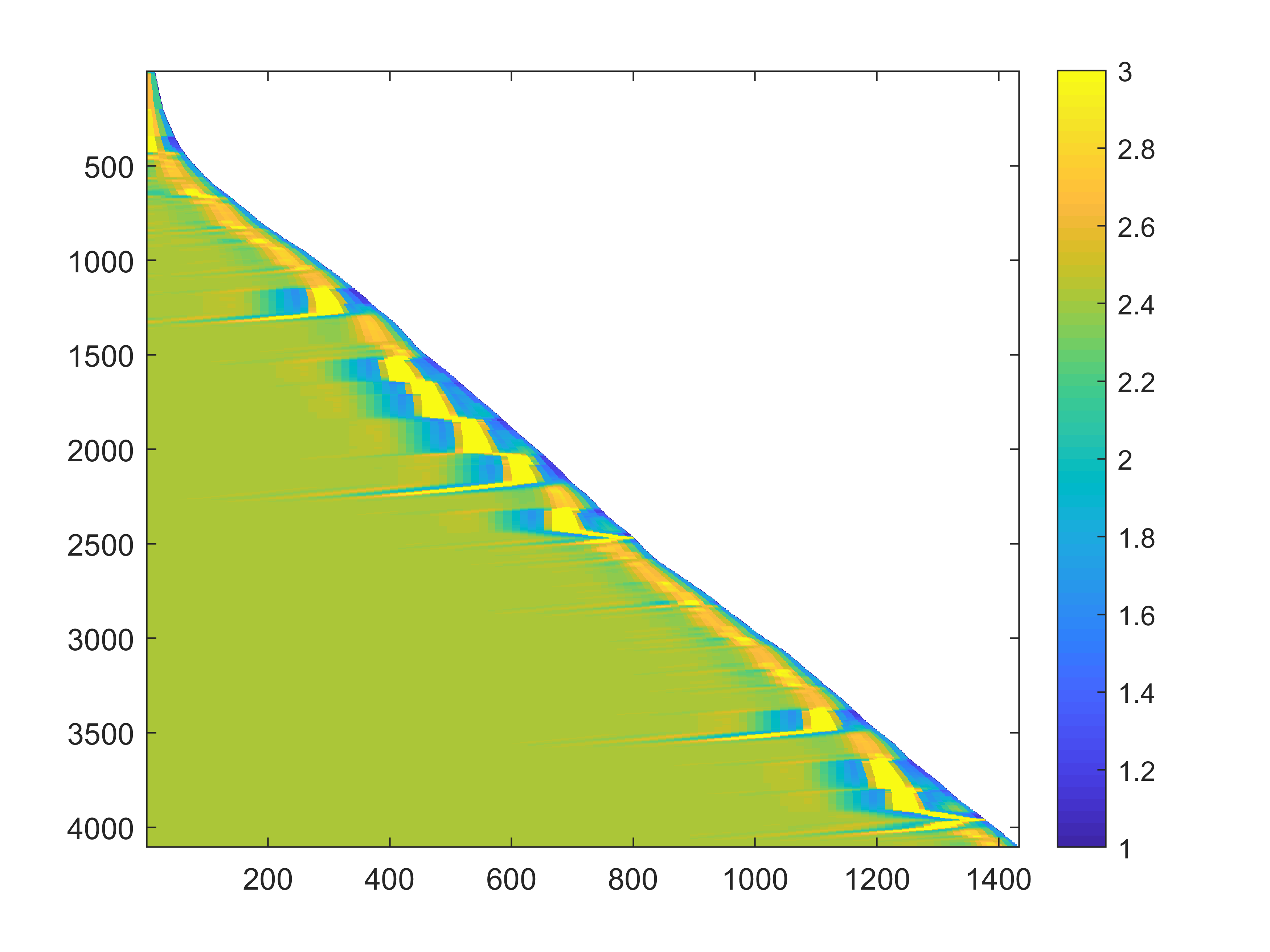

Supplement: S3 Fig — As a heatmap; same legend as Figs 2 and 3. The plots from Fig 5 were obtained by extracting intervals of 200 time units (min) from this time series with random cell division (with σ = 9.3). The graphs were generated by randomly picking start points throughout the simulation such that they would not overlap with any other interval and plotting the mean auxin over cells 6–10, for the selected 200min interval. (TIF) [file pcbi.1011646.s003.tif]
