# Supplementary material for: Fluctuations in auxin levels depend upon synchronicity of cell divisions in a one-dimensional model of auxin transport
Source: PLoS Comput Biol. 2023 Nov 30;19(11):e1011646. doi: 10.1371/journal.pcbi.1011646 (PMC10688697; doi:10.1371/journal.pcbi.1011646)
Supplement: Attachment — Submitted filename: Review_of_PCOMPBIOL_D_23_00794_.pdf [file pcbi.1011646.s012.pdf]

Review of PCOMPBIOL-D-23-00794: Fluctuations in auxin levels depend upon synchronicity of cell divisions in a one-dimensional model of auxin transpor

Andrew L. Krause, Durham University, UK*

The authors explore a model of auxin patterning in static and growing one-dimensional chains of cells. They model the cell growth and division explicitly, alongside diffusive and active transport of auxin within the cells. They make use of dynamical systems theory results, largely obtained from numerical continuation of their model, to explain synchronization (or not) of auxin oscillations as a function of parameters. A key result is that small variations in cell division time can lead to regular spatial patterns, or localized oscillations near the front. In contrast, large variations in cell division time appear to lead to irregular oscillations at the growing front. This is explained by the use of successive snaking diagrams corresponding to domains of different size, with a presumptive 'snake-jumping' between them as the growth occurs. The theory is complimented by time-lapse microscopy of an auxin reporter in *Arabidopsis* roots, showing a wide range of amplitudes of irregular oscillation, consistent with this 'snake-jumping' picture.

Overall I find the topic of the paper interesting, and the results of this study illuminating in many ways. The authors competently explained some quite technical biological and mathematical ideas, synthesizing them into a nice and captivating narrative about the role of cell growth and division in auxin dynamics. They then suggested some interesting biological implications of their theoretical results which are consistent with the experimental observations reported. Broadly the paper is well-written, and at the right technical level, though I do have a number of suggestions below to try and improve readability throughout. I also have a few scientific quibbles that I think would be worth considering, or explaining more carefully, to help improve the story being told. I would advise considering all of these comments as suggestions to think about, rather than as things that the authors must spend substantial efforts addressing in copious detail.

Major Comments

1. I feel the paper could benefit substantially from a more technical supplement than that given in the informal glossary S1 File. In particular, there were many questions I had about the numerical methods and the theory being explored that were not addressed sufficiently well in the paper (see below), but I feel could be distracting or take up too much space in the main text. As an alternative to a technical supplement, the authors could consider uploading their code to GitHub or a similar code sharing website, which supports Markdown LaTeX for very readable explanations of code and corresponding theory.

2. In the same vein as above, I find the terse descriptions of the numerical methods used quite hard to follow, whereas I find the code itself a bit unwieldy to dive into. What discretizations in time were used to produce the kymographs for instance, and what initial data were used for these? Were any convergence studies in time performed? Do the results depend much on initial auxin distributions, or do the systems relax quickly to attractors with most of the action localized to the front? I appreciate some of these questions may have answers in the cited literature on this or related models (especially the PhD Thesis [56]), but a more detailed guide would at least help orient someone trying to reproduce the results or extend the model in a different way.

3. If I understand the analysis of 'snake-jumping' correctly, the authors compute the successive fold bifurcations making up a usual snaking diagram for a fixed domain, studying only purely stationary solutions. They then interpret these in a kind of quasi-static way as the domain length changes, suggesting that the transient oscillations fall between nearby branches on the snaking diagram. I wonder if this somewhat heuristic approach can be more rigorously justified, particularly when the growth and auxin dynamics timescales are so similar (and hence one might not expect quasi-static approximations to be valid)? I am not wholly convinced that the equilibrium analysis represented by a snaking diagram is the most appropriate way to represent transients in a system with a sufficiently rapid domain growth, but I may be missing important justification for this idea.

4. Another technical issue that is unclear is if the authors considered any kind of systematic variation of parameters beyond the control parameters T and σ used. Were any ensemble studies explored with different parameters or initial

*By signing this review, I waive my right to anonymity.

data to determine how robust the transient dynamics were? Are the snaking diagrams, and the time-dependent behaviours observed in the growing system, structurally stable to modest perturbations in the system parameters?

5. The authors state in Line 435 that continuum approximations are unpractical. Can this statement be justified more carefully, either by a Knudsen-number type argument (i.e. that there are too few cells for the validity of such an approximation), or that a mean-field limit is not accessible for a more technical reason?

6. Finally, the last few sentences of the conclusion are a bit lackluster. The final sentence of the abstract makes an interesting claim, but this seemed to not be the punchline of the conclusion (or if it was I did not follow the somewhat vague reasoning in the last part of the conclusion). Perhaps the authors could consider more carefully making precise conjectures/hypotheses about the role of auxin in observed oscillations in plants? This seems like a worthwhile takeaway from the study that could be elaborated on a bit more.

Below are minor comments, mostly typographical/presentation. As there are a number of small typos that I found, I would encourage the authors to carefully reread the manuscript to minimize such errors, particularly missing articles between words.

Minor Comments/Typos

7. The author summary is missing a few articles and in general could be more clear in its meaning I think (though I appreciate this is difficult to do in a concise way). For example, the sentence “More physiological conditions including variations in the timing of cell divisions leads to much less temporal regularity in auxin variations,” is a bit of a mouthful and hard to interpret as written. I think the authors mean “Physiological variations in the timing of cell divisions leads to reduced temporal regularity in auxin oscillations” or something to this effect.

8. The authors cite [20] referring to growing domains and reaction-diffusion models, though I believe this paper has little do with growth. More relevant papers by these authors might be [Seirin Lee et al., 2011, Krause et al., 2019, Van Gorder et al., 2021, Krause et al., 2023], though it is by no means necessary that the authors cite these, as they already have several good examples mentioned (and the review [21] more exhaustively lists these). The last paper above may be relevant as an example of concentration-dependent growth, though only in the case of continuum models with Fickian transport.

9. Line 99: “The use of the term ‘snaking diagram’ to refer to these folds.” is an incomplete sentence. I think a few words are missing here.

10. The term “auxin variations” is used in several places. I interpret this to mean temporal changes, typically temporal oscillations, but perhaps the authors could clarify earlier in the paper precisely what they mean by this term?

11. Line 171: missing an article; I think it should say “is used as ‘the’ main bifurcation line” instead.

12. Line 255: missing an “if” or similar rephrasing.

13. Line 258: delete the extra period before the citation.

14. Line 260: missing ‘of’ or similar rephrasing.

15. Line 290: missing ‘the’ or similar rephrasing.

16. Lines 309-317 contain two sentences that are repeated verbatim twice.

17. Lines 325-326: The last sentence of this paragraph is completely unintelligible to me. What exactly did it mean to convey?

18. There are a few places where equality typesetting is inconsistent, such as in the caption of Fig 5 where the spacing after the equality following σ seems incongruent.

19. Line 400: There is a change of person (tense); use of “one” rather than “we” with the latter more common throughout the manuscript. Consider just being consistent.

20. Line 467: ‘enforced’ should be ‘enforce’ I think.

21. I feel that many of the Figures are a bit low quality, and could be made much easier to read. This may be an issue of how they were enlarged in the editorial system. I would just recommend ensuring that the lines are suitably thick, and axes labels are sufficiently large and legible in the finalized manuscript.

References

[Krause et al., 2019] Krause, A. L., Ellis, M. A., and Van Gorder, R. A. (2019). Influence of curvature, growth, and anisotropy on the evolution of Turing patterns on growing manifolds. *Bulletin of Mathematical Biology*, 81(3):759–799.

[Krause et al., 2023] Krause, A. L., Gaffney, E. A., and Walker, B. J. (2023). Concentration-dependent domain evolution in reaction–diffusion systems. *Bulletin of Mathematical Biology*, 85(2):14.

[Seirin Lee et al., 2011] Seirin Lee, S., Gaffney, E. A., and Baker, R. E. (2011). The dynamics of turing patterns for morphogen-regulated growing domains with cellular response delays. *Bulletin of mathematical biology*, 73:2527–2551.

[Van Gorder et al., 2021] Van Gorder, R. A., Klika, V., and Krause, A. L. (2021). Turing conditions for pattern forming systems on evolving manifolds. *Journal of Mathematical Biology*, 82(4).
