# Supplementary material for: Fluctuations in auxin levels depend upon synchronicity of cell divisions in a one-dimensional model of auxin transport
Source: PLoS Comput Biol. 2023 Nov 30;19(11):e1011646. doi: 10.1371/journal.pcbi.1011646 (PMC10688697; doi:10.1371/journal.pcbi.1011646)
Supplement: Attachment — Submitted filename: ResponseLetter.pdf [file pcbi.1011646.s013.pdf]

School of Mathematical Sciences
University of Nottingham
University Park
Nottingham
NG7 2RD

+44 (0)115 951 4943
Etienne.Farcot@nottingham.ac.uk

14 September 2023

Manuscript PCOMPBIOL-D-23-00794: ‘Fluctuations in auxin levels depend upon synchronicity of cell divisions in a one-dimensional model of auxin transport’ -

Dear Editors and Reviewers,

We first would like to thank you for the thorough reviews. They have greatly helped achieve a clearer and improved presentation. Reviewers 1 and 2 have structured their reviews in major/minor comments. We first discuss all major comments, leaving discussion on minor comments as an appendix to this letter.

Reviewer 1:

The authors use a combination of mathematical modelling and experimental data to analyse potential auxin patterns at varying auxin transport rates. This analysis comprises a static scenario, one with deterministic growth and one with variations in the timing and divisions of cell volumes. As this manuscript represents the first analysis that includes cell division, this provides interesting insight into auxin patterns as auxin signalling is tightly interwoven with growth processes. The inclusion of variability in the cell division process makes it particularly interesting.

We thank Reviewer 1 for this positive introduction.

However, I currently see some major points where the manuscript can be improved:

1. As the authors rely on one model for auxin transport throughout the manuscript, the paper would benefit greatly from an overview figure detailing the model reactions and layout. It would also be helpful to highlight the differences between the different scenarios considered (static, deterministic growth and fluctuations in cell division).
Thank you for this useful suggestion, which improves clarity. We have created a new figure (now Fig6) which appears in Material and Methods where the model is described.

2. The authors begin with an analysis of a static system showing the different potential auxin patterns at different auxin transport values. The model structure is such that a single cell file is considered, which could “represent a single file of cells within a root” (l. 123) and where every cell has the same size. However, when we look at the actual plant root, this is not the case (see for example van Esse et al. 2011 or Beemster & Baskin 1998). Instead, cells sizes increase a lot as we get further away from the meristematic zone. With a cell file consisting of 128 cells, where one end represents the connection to the mature root, different cell sizes definitely occur and might impact the observable auxin patterns. By only considering one cell size for this analysis, it limits the scope of the model and the conclusions one can

draw from it. As the single cell file is a rather simple accounting for different cell sizes throughout the file should not be too difficult and would increase the informative value of this analysis.

Thank you for this interesting suggestion. It is correct that our section on the static model only considered a uniform cell size and that this is a limitation. We have created a new figure (now included within supplementary S2_Fig) showing a snaking diagram for a tissue in which cells have a non-uniform size. More specifically the cell sizes represent a more realistic progression from smaller cells at the tip to cells approximately 10 times larger, in line with data in Beemster & Baskin 1998. This new simulation confirms the importance of the underlying tissue geometry for auxin patterns: significant differences in cell volumes tend to reduce multistability and force the system in a more limited number of configurations for any fixed value of T. However, it remains true that changes of patterns can be created from growth/divisions and the general principles presented in the paper are still as described previously. We have added more text (l. 167-178) and a paragraph in the discussion (penultimate paragraph), as the point raised here is important.

3. The authors made the decision to model growth at a constant rate (g) up to a maximal volume of 4/3 L3, where cells either divide if they are in the respective root region or stop growing altogether. While the maximal volume of 4/3 L3 as division threshold lines up nicely with cell sizes in the root, the choice of growth rate and Vmax as final cell volume represent important modelling decisions / simplifications: When we look at the actual growth rates in the root, we see that the actual growth rate varies strongly depending on the cell's position in the root and that cells grow well beyond 4/3 L3 (e.g. Beemster & Baskin 1998). While all models include certain simplifications and the decision to describe growth like this is understandable, it should be clearly mentioned in the introduction of the growth model. The fact that these simplifications can affect the model behaviour and – as a consequence – also the conclusions we can draw from the simulations should also be addressed in the discussion.

This is a fair point and we have included some words of caution in the model presentation (lines 188-189) as well as in the discussion (penultimate paragraph). We appreciate this remark, which leads to a more objective discussion and leads to interesting suggestions for future work.

4. Regarding the figures: Some figures are missing the axis labels, some are missing the units of what is shown, some subfigures are labelled at the bottom of the subfigure, some at the top. Please correct the figure format so that all figures include axis labels and the respective units (even if its arbitrary units).

We have revised all figures to improve quality and readability.

5. While I understand the difficulties in matching the DII:Venus measurements to the simulated auxin dynamics, at least one example where simulated and experimental results are shown in one figure together would make this comparison much easier for the reader to follow.

We have incorporated such an image within Fig5, where panels (b) and (d) are now overlayed with experimental data from Fig4.

Reviewer 2:

The authors explore a model of auxin patterning in static and growing one-dimensional chains of cells. They model the cell growth and division explicitly, alongside diffusive and active transport of auxin within the cells. They make use of dynamical systems theory results, largely obtained from numerical continuation of their model, to explain synchronization (or not) of auxin oscillations as a function of parameters. A key result is that small variations in cell division time can lead to regular spatial patterns, or localized oscillations near the front. In contrast, large variations in cell division time appear to lead to

irregular oscillations at the growing front. This is explained by the use of successive snaking diagrams corresponding to domains of different size, with a presumptive 'snake-jumping' between them as the growth occurs. The theory is complimented by time-lapse microscopy of an auxin reporter in Arabidopsis roots, showing a wide range of amplitudes of irregular oscillation, consistent with this 'snake-jumping' picture.
Overall I find the topic of the paper interesting, and the results of this study illuminating in many ways. The authors competently explained some quite technical biological and mathematical ideas, synthesizing them into a nice and captivating narrative about the role of cell growth and division in auxin dynamics. They then suggested some interesting biological implications of their theoretical results which are consistent with the experimental observations reported. Broadly the paper is well-written, and at the right technical level, though I do have a number of suggestions below to try and improve readability throughout. I also have a few scientific quibbles that I think would be worth considering, or explaining more carefully, to help improve the story being told. I would advise considering all of these comments as suggestions to think about, rather than as things that the authors must spend substantial efforts addressing in copious detail.
Thank you for this positive overview.

1. I feel the paper could benefit substantially from a more technical supplement than that given in the informal glossary S1 File. In particular, there were many questions I had about the numerical methods and the theory being explored that were not addressed sufficiently well in the paper (see below), but I feel could be distracting or take up too much space in the main text. As an alternative to a technical supplement, the authors could consider uploading their code to GitHub or a similar code sharing website, which supports Markdown LaTeX for very readable explanations of code and corresponding theory.
It is true that our description of the numerical methods remains superficial. Being conscious of the multidisciplinary readership of PLoS Computational Biology, we wanted to avoid as many technicalities as possible. However, we agree that for sake of reproducibility all methods should be described in full detail. Note that we have not created any new numerical methods in this paper and besides standard Matlab routines, for continuation we rely on a previous implementation by one of the authors cited as reference [51] (previously [44]). This reference includes the code, a complete tutorial and technical references for the numerical continuation methods. We now point at this reference more clearly in the description of S2_File (the code that was used specifically for the present paper, see also next comment).

2. In the same vein as above, I find the terse descriptions of the numerical methods used quite hard to follow, whereas I find the code itself a bit unwieldy to dive into. What discretizations in time were used to produce the kymographs for instance, and what initial data were used for these? Were any convergence studies in time performed? Do the results depend much on initial auxin distributions, or do the systems relax quickly to attractors with most of the action localized to the front? I appreciate some of these questions may have answers in the cited literature on this or related models (especially the PhD Thesis [56]), but a more detailed guide would at least help orient someone trying to reproduce the results or extend the model in a different way.
We agree that our previous text did not provide sufficient details and that the code was difficult to navigate. We have restructured the code to make it more readable and have added some necessary explanations at the end of the *Implementing growth* section in the methods to address the point about the kymographs (l.514-521), as well as in the caption for S2_File (where the code is located).

3. If I understand the analysis of 'snake-jumping' correctly, the authors compute the successive fold bifurcations making up a usual snaking diagram for a fixed domain, studying only purely stationary solutions. They then interpret these in a kind of quasi-static way as the domain length changes, suggesting that the transient oscillations fall between nearby branches on the snaking diagram. I wonder if this

somewhat heuristic approach can be more rigorously justified, particularly when the growth and auxin dynamics timescales are so similar (and hence one might not expect quasi-static approximations to be valid)? I am not wholly convinced that the equilibrium analysis represented by a snaking diagram is the most appropriate way to represent transients in a system with a sufficiently rapid domain growth, but I may be missing important justification for this idea.

Thank you for raising this important point. Our approach to rely on steady states, in a context which is inherently transient is definitely heuristic rather that grounded in a completely formal justification. However, we believe this approximation is consistent with the known timescales for this system, though this was not made entirely explicit in our first submission. We have now included an additional paragraph in introduction (l.52-57); we repeat the main point here: at the cellular scale the motion of auxin driven by active transporters is most likely very rapid compared to growth, with a typical speed of 10mm/h allowing to go through an entire meristem in about a minute or less. Auxin signalling is equally fast. The timescales which are comparable are those of cell division and auxin oscillations, which was the original motivation for this work.

4. Another technical issue that is unclear is if the authors considered any kind of systematic variation of parameters beyond the control parameters T and σ used. Were any ensemble studies explored with different parameters or initial data to determine how robust the transient dynamics were? Are the snaking diagrams, and the time-dependent behaviours observed in the growing system, structurally stable to modest perturbations in the system parameters?

We have not performed a systematic analysis of the robustness of the model to parameter changes. Neither have we systematically considered ensembles of initial conditions to assess the robustness of transients. This is not to say we have no evidence of robustness, but that this was assessed in a non-systematic, exploratory way. Indeed, in preliminary investigations leading to this paper, we have performed extensive numerical studies, for various parameter choices and initial conditions. Though informal, this manual exploration has convinced us of the robustness of the main claims made in the paper. We have added a comment in the discussion to clarify this empirical approach and how future work could overcome its lack of comprehensiveness (paragraph starting l. 383).

5. The authors state in Line 435 that continuum approximations are unpractical. Can this statement be justified more carefully, either by a Knudsen-number type argument (i.e. that there are too few cells for the validity of such an approximation), or that a mean-field limit is not accessible for a more technical reason?

The main reason behind this statement is not a restriction on the relation between spatial scales and cell numbers. It is the fact that the anisotropic, auxin dependent nature of PIN distribution within cells leads to continuum approximation which are typically nonlinear and/or involve high order terms. Furthermore, they typically involve a quasi-steady approximation on the dynamics of PINs, ultimately removing them from equations. The mathematically non-straightforward derivation, and the fact that PIN are experimentally easier to observe than auxin, has made such approximations uncommon in the literature. To our knowledge there is only one detailed example in the literature, and we have modified the paragraph mentioned in this comment to include the main reference and justify our claim more carefully.

6. Finally, the last few sentences of the conclusion are a bit lackluster. The final sentence of the abstract makes an interesting claim, but this seemed to not be the punchline of the conclusion (or if it was I did not follow the somewhat vague reasoning in the last part of the conclusion). Perhaps the authors could consider more carefully making precise conjectures/hypotheses about the role of auxin in observed oscillations in plants? This seems like a worthwhile takeaway from the study that could be elaborated on a bit more.

We agree that our previous final paragraph was rather underwhelming and have updated the final section in this revised manuscript.

Reviewer 3:

This manuscript reports on the effects of cell growth and divisions on the dynamics of auxin patterning, using a combination of mathematical modelling and experimental observations. The conclusion is that steady patterns in auxin distribution resulting in a geometric curve with successive folds, as predicted by the model and under a regime of rhythmic cell divisions, become easily disturbed which the authors designated as 'snake-jumping'. High level of synchronization between cell divisions are thus a prerequisite to obtain oscillatory patterns in auxin distribution. I believe this is a valuable piece of work which underlines the importance of the cell division behavior in plant tissues as a major parameter to include in all further work on auxin distribution dynamics and will contribute to a better understanding of developmental processes that rely on the spatial distribution of auxin (and there are many).
I am not well placed to comment on the quality of the calculations in the modelling approach, but I made a few points that the authors should take into account to avoid overstatements based on generalizations taken from literature.

Thank you for the appreciation of our work.

1. The assumption that cell divisions in the meristem are characterized by a great level of variability might not be necessarily true. Depending on the zone and tissue, synchronized cell divisions have been reported. Divisions within in the transit amplifying cells in the meristem appeared to be homogenous (Week-long imaging of cell divisions in the Arabidopsis root meristem by Ramin Rahni & Kenneth D. Birnbaum,Plant Methods volume 15, Article number: 30 (2019). Oscillations might therefore occur in synchronized populations of cells in the root.
This is an important point. The deterministic nature of cell division in our model is a necessary simplification, as we need a mechanism to drive growth and division. To improve on this would require a detailed quantitative model of cell division that is not currently available. We have edited the text where we describe the variable template growth to highlight this generalization (l. 531-534) and we also comment again in the discussion.

2. The experimental data using the DII Venus marker did not show any sign of synchronization in the auxin response/accumulation.
I believe the experimental approach used by the authors was inappropriate to detect this for the following reasons:
- Researchers trying to measure oscillation behavior focus on a well-defined zone in the root (probably where cell divisions have a higher level of synchronization), which was not taken into account in the experimental set-up of the present study. Furthermore, the auxin maxima are not occurring in cortex cells which were the cells exclusively used here. DR5-luciferase does not allow to situate the auxin maxima in one cell type or another but comparative analysis with DR5-Venus shows absence of auxin accumulation in the cortex while it is all happening in the vascular bundle.
- When individual roots are compared without determining a time 0 to start the recordings, the probability to see oscillations is nihil. Therefore, typically recordings are made starting from the first peak in auxin response and measuring amplitude and period of the following peaks.

- Roots grow vertically and reorienting them in a horizontal position to make recordings is a drastic action certainly when it comes to auxin distribution patterns. According the M&M this has not been taken into account in the experimental observations.
In conclusion, I am not asking to repeat experiments only to include some caution while referring to the real situation.
We thank the reviewer for raising this important point. We have re-analysed the same data set by quantifying DII response in the stele. This is included in a revised figure 4. The results are in line with the previous quantification where we do not see any clear oscillatory behaviour.
We made recordings for a period of 18h, and therefore if there were oscillations we would expect to see them. We acknowledge that the roots may not be synchronised. Whilst we see the advantage of aligning them based on the first peak in auxin, this was not feasible because many roots did not show any clear auxin peak. We explored the data by trying to align the highest point, but no pattern was clear enough to be reported in the paper.
The reviewer is correct that we had to re-orientate plants during imaging. This was approximately 1 minute or less per plant and is now described in the materials and methods; we have also added a word of caution to the discussion (l. 398).

We believe that these changes address points that were raised. We remain at your disposal should there be any question, or request for additional clarifications or manuscript amendments.

Sincerely yours,

Etienne Farcot, PhD
Associate Professor
School of Mathematical Sciences
University of Nottingham, UK

Appendix: Minor comments

Reviewer 1:
At several points throughout the manuscript there are missing units of parameters or concentrations (e.g. line 148 "a 10-18 change in auxin concentration"). Please correct this throughout the text and in the figures.
We meant the 10^{-18} to be a percentage, arbitrary but indicative of infinitesimal variations. We have corrected this a reviewed the document to include units wherever they were missing.

In the literature summary in the introduction, some of the references are placed counterintuitively and are missing where I would expect them:
- Lines 43-44: which studies?
- Lines 93-95: where is this other analysis published?
- Lines 125-126: which previous studies?
We have corrected all three issues above.

Also, a couple of relevant studies are missing in the introduction including:
- Allen HR, Ptashnyk M (2020) Mathematical Modelling of Auxin Transport in Plant Tissues: Flux Meets Signalling and Growth. Bulletin of Mathematical Biology

- Twycross J et al. (2010) Stochastic and deterministic multiscale models for systems biology: an auxin-transport case study. BMC Systems Biology.
The two references have been added.

Finally, some minor details:
- The inclusion of S1 File is a nice way of providing the basic terminology of dynamical system theory. However, an equation of the L2 norm is calculated would not be amiss.
The formula has been added in the figure caption in S1_file
- Line 318: "[…] time series, see." See what?
This was meant to refer to the figure; now corrected.

Reviewer 2:

7. *The author summary is missing a few articles and in general could be more clear in its meaning I think (though I appreciate this is difficult to do in a concise way). For example, the sentence "More physiological conditions including variations in the timing of cell divisions leads to much less temporal regularity in auxin variations," is a bit of a mouthful and hard to interpret as written. I think the authors mean "Physiological variations in the timing of cell divisions leads to reduced temporal regularity in auxin oscillations" or something to this effect.*
   Many thanks, we have used your excellent suggestion as it is.

8. *The authors cite [20] referring to growing domains and reaction-diffusion models, though I believe this paper has little do with growth. More relevant papers by these authors might be [Seirin Lee et al., 2011, Krause et al., 2019, Van Gorder et al., 2021, Krause et al., 2023], though it is by no means necessary that the authors cite these, as they already have several good examples mentioned (and the review [21] more exhaustively lists these). The last paper above may be relevant as an example of concentration-dependent growth, though only in the case of continuum models with Fickian transport.*
   Thank you for providing us with more relevant literature, we have replaced reference [20], which indeed is not focussed on growth, nut the three suggestions above which all are relevant.

9. *Line 99: "The use of the term 'snaking diagram' to refer to these folds." is an incomplete sentence. I think a few words are missing here.*
   We have reintroduced a word which had been accidentally deleted.

10. *The term "auxin variations" is used in several places. I interpret this to mean temporal changes, typically temporal oscillations, but perhaps the authors could clarify earlier in the paper precisely what they mean by this term?*
    We have add the the adjective "temporal" to clarify.

    All edits required below have been implemented. Re: figures, see also response to point 4 from Reviewer 1.
11. *Line 171: missing an article; I think it should say "is used as 'the' main bifurcation line" instead.*
12. *Line 255: missing an "if" or similar rephrasing.*
13. *Line 258: delete the extra period before the citation.*
14. *Line 260: missing 'of' or similar rephrasing.*
15. *Line 290: missing 'the' or similar rephrasing.*
16. *Lines 309-317 contain two sentences that are repeated verbatim twice.*

17. *Lines 325-326: The last sentence of this paragraph is completely unintelligible to me. What exactly did it mean to convey?*
18. *There are a few places where equality typesetting is inconsistent, such as in the caption of Fig 5 where the spacing after the equality following σ seems incongruent.*
19. *Line 400: There is a change of person (tense); use of “one” rather than “we” with the latter more common throughout the manuscript. Consider just being consistent.*
20. *Line 467: ‘enforced’ should be ‘enforce’ I think.*
21. *I feel that many of the Figures are a bit low quality, and could be made much easier to read. This may be an issue of how they were enlarged in the editorial system. I would just recommend ensuring that the lines are suitably thick, and axes labels are sufficiently large and legible in the finalized manuscript.*
