# Supplementary material for: Fluctuations in auxin levels depend upon synchronicity of cell divisions in a one-dimensional model of auxin transport
Source: PLoS Comput Biol. 2023 Nov 30;19(11):e1011646. doi: 10.1371/journal.pcbi.1011646 (PMC10688697; doi:10.1371/journal.pcbi.1011646)
Supplement: Attachment — Submitted filename: ResponseLetter2.pdf [file pcbi.1011646.s014.pdf]

University of Nottingham
UK | CHINA | MALAYSIA

School of Mathematical Sciences
University of Nottingham
University Park
Nottingham
NG7 2RD

+44 (0)115 951 4943
Etienne.Farcot@nottingham.ac.uk

30 October 2023

Manuscript PCOMPBIOL-D-23-00794R1: ‘Fluctuations in auxin levels depend upon synchronicity of cell divisions in a one-dimensional model of auxin transport’ -

Dear Editors and Reviewers,

Thank you again for reviewing this revised manuscript with care. All final comments were requests for syntactic improvements and corrections of typos. For completeness, the list of required changes is reproduced below. We have implemented all these corrections apart from the l.557 from Reviewer 2, which was an artifact of the changes highlight applied to our revision.
We remain at your disposal for any further clarification or correction.

Sincerely yours,

Etienne Farcot, PhD

Associate Professor
School of Mathematical Sciences
University of Nottingham, UK

Reviewer #1: *All of my major concerns regarding the previous version have been sufficiently addressed.*
I only have a few minor comments:
I appreciate the inclusion of Fig. 6 in the materials and methods section. It might also be worthwhile to mention the figure in the results section, where the model is introduced. Maybe something like “See Materials and methods for equations and overview figure” in line 129.
ll. 179 “This lower level of multistability is consistent with experimental observations, which do not typically report high numbers of auxin response maxima.” -> reference(s)?
l. 247 “The Case 2” -> “The case 2 […]”
ll. 253 This sentence is rather difficult to read: “One observation that can be made is that for intermediate values of T, near the tip of the domain auxin concentration oscillates.”
Maybe: “One observation that can be made is that the auxin concentration oscillates near the tip of the domain for intermediate values of T.”
Or at least a second comma: “One observation that can be made is that for intermediate values of T, near the tip of the domain, auxin concentration oscillates.”

Reviewer #2: *The authors have done an excellent job in polishing their manuscript, and writing a fascinating story combining dynamical systems modelling and important biological insights. Below are some minor comments that they may consider, but I have no reservations for recommending this manuscript to be published. Well done!*
Line 175: The notation "(10X)" could be spelled out to be more precise as "(10 times)".
Line 557: A space is missing between the . and the word "Plants".
Line 579: "Fewer" would be better than "less".

Reviewer #3: *The authors have taken my comments into account and I am satisfied with their answers and with the textual changes in the new version of their manuscript.*
