# Supplementary material for: Fluctuations in auxin levels depend upon synchronicity of cell divisions in a one-dimensional model of auxin transport
Source: PLoS Comput Biol. 2023 Nov 30;19(11):e1011646. doi: 10.1371/journal.pcbi.1011646 (PMC10688697; doi:10.1371/journal.pcbi.1011646)
Supplement: Peer review history

# Peer review: Decision Letter 0

**DOI:** 10.1371/journal.pcbi.1011646.r001

**Authors:** Pedro Mendes, Roeland M.H. Merks

27 Jun 2023

Dear Dr. Farcot,

Thank you very much for submitting your manuscript "Fluctuations in auxin levels depend upon synchronicity of cell divisions in a one-dimensional model of auxin transport" for consideration at PLOS Computational Biology.

As with all papers reviewed by the journal, your manuscript was reviewed by members of the editorial board and by several independent reviewers. In light of the reviews (below this email), we would like to invite the resubmission of a significantly-revised version that takes into account the reviewers' comments.

We cannot make any decision about publication until we have seen the revised manuscript and your response to the reviewers' comments. Your revised manuscript is also likely to be sent to reviewers for further evaluation.

When you are ready to resubmit, please upload the following:

[1] A letter containing a detailed list of your responses to the review comments and a description of the changes you have made in the manuscript. Please note while forming your response, if your article is accepted, you may have the opportunity to make the peer review history publicly available. The record will include editor decision letters (with reviews) and your responses to reviewer comments. If eligible, we will contact you to opt in or out.

[2] Two versions of the revised manuscript: one with either highlights or tracked changes denoting where the text has been changed; the other a clean version (uploaded as the manuscript file).

Important additional instructions are given below your reviewer comments.

Please prepare and submit your revised manuscript within 60 days. If you anticipate any delay, please let us know the expected resubmission date by replying to this email. Please note that revised manuscripts received after the 60-day due date may require evaluation and peer review similar to newly submitted manuscripts.

Thank you again for your submission. We hope that our editorial process has been constructive so far, and we welcome your feedback at any time. Please don't hesitate to contact us if you have any questions or comments.

Sincerely,

Roeland M.H. Merks, Ph.D

Academic Editor

PLOS Computational Biology

Pedro Mendes

Section Editor

PLOS Computational Biology

***********************

Reviewer's Responses to Questions

**Comments to the Authors:**

**Please note here if the review is uploaded as an attachment.**

Reviewer #1: Review of 'Fluctuations in auxin levels depend upon synchronicity of cell divisions in a one-dimensional model of auxin transport'

The authors use a combination of mathematical modelling and experimental data to analyse potential auxin patterns at varying auxin transport rates. This analysis comprises a static scenario, one with deterministic growth and one with variations in the timing and divisions of cell volumes. As this manuscript represents the first analysis that includes cell division, this provides interesting insight into auxin patterns as auxin signalling is tightly interwoven with growth processes. The inclusion of variability in the cell division process makes it particularly interesting.

However, I currently see some major points where the manuscript can be improved:

1. As the authors rely on one model for auxin transport throughout the manuscript, the paper would benefit greatly from an overview figure detailing the model reactions and layout. It would also be helpful to highlight the differences between the different scenarios considered (static, deterministic growth and fluctuations in cell division).

2. The authors begin with an analysis of a static system showing the different potential auxin patterns at different auxin transport values. The model structure is such that a single cell file is considered, which could “represent a single file of cells within a root” (l. 123) and where every cell has the same size. However, when we look at the actual plant root, this is not the case (see for example van Esse et al. 2011 or Beemster & Baskin 1998). Instead, cells sizes increase a lot as we get further away from the meristematic zone. With a cell file consisting of 128 cells, where one end represents the connection to the mature root, different cell sizes definitely occur and might impact the observable auxin patterns. By only considering one cell size for this analysis, it limits the scope of the model and the conclusions one can draw from it. As the single cell file is a rather simple accounting for different cell sizes throughout the file should not be too difficult and would increase the informative value of this analysis.

3. The authors made the decision to model growth at a constant rate (g) up to a maximal volume of 4/3 L3, where cells either divide if they are in the respective root region or stop growing altogether. While the maximal volume of 4/3 L3 as division threshold lines up nicely with cell sizes in the root, the choice of growth rate and Vmax as final cell volume represent important modelling decisions / simplifications: When we look at the actual growth rates in the root, we see that the actual growth rate varies strongly depending on the cell’s position in the root and that cells grow well beyond 4/3 L3 (e.g. Beemster & Baskin 1998). While all models include certain simplifications and the decision to describe growth like this is understandable, it should be clearly mentioned in the introduction of the growth model. The fact that these simplifications can affect the model behaviour and – as a consequence – also the conclusions we can draw from the simulations should also be addressed in the discussion.

4. Regarding the figures: Some figures are missing the axis labels, some are missing the units of what is shown, some subfigures are labelled at the bottom of the subfigure, some at the top. Please correct the figure format so that all figures include axis labels and the respective units (even if its arbitrary units).

5. While I understand the difficulties in matching the DII:Venus measurements to the simulated auxin dynamics, at least one example where simulated and experimental results are shown in one figure together would make this comparison much easier for the reader to follow.

Additional comments:

At several points throughout the manuscript there are missing units of parameters or concentrations (e.g. line 148 “a 10-18 change in auxin concentration”). Please correct this throughout the text and in the figures.

In the literature summary in the introduction, some of the references are placed counterintuitively and are missing where I would expect them:

- Lines 43-44: which studies?

- Lines 93-95: where is this other analysis published?

- Lines 125-126: which previous studies?

Also, a couple of relevant studies are missing in the introduction including:

- Allen HR, Ptashnyk M (2020) Mathematical Modelling of Auxin Transport in Plant Tissues: Flux Meets Signalling and Growth. Bulletin of Mathematical Biology

- Twycross J et al. (2010) Stochastic and deterministic multiscale models for systems biology: an auxin-transport case study. BMC Systems Biology.

Finally, some minor details:

- The inclusion of S1 File is a nice way of providing the basic terminology of dynamical system theory. However, an equation of the L2 norm is calculated would not be amiss.

- Line 318: “[…] time series, see.” See what?

Reviewer #2: Please see attached comments.

Reviewer #3: This manuscript reports on the effects of cell growth and divisions on the dynamics of auxin patterning, using a combination of mathematical modelling and experimental observations. The conclusion is that steady patterns in auxin distribution resulting in a geometric curve with successive folds, as predicted by the model and under a regime of rhythmic cell divisions, become easily disturbed which the authors designated as ’snake-jumping’. High level of synchronization between cell divisions are thus a prerequisite to obtain oscillatory patterns in auxin distribution. I believe this is a valuable piece of work which underlines the importance of the cell division behavior in plant tissues as a major parameter to include in all further work on auxin distribution dynamics and will contribute to a better understanding of developmental processes that rely on the spatial distribution of auxin (and there are many).

I am not well placed to comment on the quality of the calculations in the modelling approach, but I made a few points that the authors should take into account to avoid overstatements based on generalizations taken from literature.

1. The assumption that cell divisions in the meristem are characterized by a great level of variability might not be necessarily true. Depending on the zone and tissue, synchronized cell divisions have been reported. Divisions within in the transit amplifying cells in the meristem appeared to be homogenous (Week-long imaging of cell divisions in the Arabidopsis root meristem by Ramin Rahni & Kenneth D. Birnbaum,Plant Methods volume 15, Article number: 30 (2019). Oscillations might therefore occur in synchronized populations of cells in the root.

2. The experimental data using the DII Venus marker did not show any sign of synchronization in the auxin response/accumulation.

I believe the experimental approach used by the authors was inappropriate to detect this for the following reasons:

- Researchers trying to measure oscillation behavior focus on a well-defined zone in the root (probably where cell divisions have a higher level of synchronization), which was not taken into account in the experimental set-up of the present study. Furthermore, the auxin maxima are not occurring in cortex cells which were the cells exclusively used here. DR5-luciferase does not allow to situate the auxin maxima in one cell type or another but comparative analysis with DR5-Venus shows absence of auxin accumulation in the cortex while it is all happening in the vascular bundle.

- When individual roots are compared without determining a time 0 to start the recordings, the probability to see oscillations is nihil. Therefore, typically recordings are made starting from the first peak in auxin response and measuring amplitude and period of the following peaks.

- Roots grow vertically and reorienting them in a horizontal position to make recordings is a drastic action certainly when it comes to auxin distribution patterns. According the M&M this has not been taken into account in the experimental observations.

In conclusion, I am not asking to repeat experiments only to include some caution while referring to the real situation.

**********

**Have the authors made all data and (if applicable) computational code underlying the findings in their manuscript fully available?**

The PLOS Data policy requires authors to make all data and code underlying the findings described in their manuscript fully available without restriction, with rare exception (please refer to the Data Availability Statement in the manuscript PDF file). The data and code should be provided as part of the manuscript or its supporting information, or deposited to a public repository. For example, in addition to summary statistics, the data points behind means, medians and variance measures should be available. If there are restrictions on publicly sharing data or code —e.g. participant privacy or use of data from a third party—those must be specified.

Reviewer #1: Yes

Reviewer #2: Yes

Reviewer #3: Yes

**********

PLOS authors have the option to publish the peer review history of their article (what does this mean?). If published, this will include your full peer review and any attached files.

If you choose “no”, your identity will remain anonymous but your review may still be made public.

**Do you want your identity to be public for this peer review?** For information about this choice, including consent withdrawal, please see our Privacy Policy.

Reviewer #1: No

Reviewer #2: **Yes: **Andrew L. Krause

Reviewer #3: No

Figure Files:

While revising your submission, please upload your figure files to the Preflight Analysis and Conversion Engine (PACE) digital diagnostic tool, https://pacev2.apexcovantage.com. PACE helps ensure that figures meet PLOS requirements. To use PACE, you must first register as a user. Then, login and navigate to the UPLOAD tab, where you will find detailed instructions on how to use the tool. If you encounter any issues or have any questions when using PACE, please email us at figures@plos.org.

Data Requirements:

Please note that, as a condition of publication, PLOS' data policy requires that you make available all data used to draw the conclusions outlined in your manuscript. Data must be deposited in an appropriate repository, included within the body of the manuscript, or uploaded as supporting information. This includes all numerical values that were used to generate graphs, histograms etc.. For an example in PLOS Biology see here: http://www.plosbiology.org/article/info%3Adoi%2F10.1371%2Fjournal.pbio.1001908#s5.

Reproducibility:

To enhance the reproducibility of your results, we recommend that you deposit your laboratory protocols in protocols.io, where a protocol can be assigned its own identifier (DOI) such that it can be cited independently in the future. Additionally, PLOS ONE offers an option to publish peer-reviewed clinical study protocols. Read more information on sharing protocols at https://plos.org/protocols?utm_medium=editorial-email&utm_source=authorletters&utm_campaign=protocols

Attachment Submitted filename: Review_of_PCOMPBIOL_D_23_00794_.pdf Click here for additional data file.

# Author comment: Author response to Decision Letter 0

**DOI:** 10.1371/journal.pcbi.1011646.r002

19 Sep 2023

Attachment Submitted filename: ResponseLetter.pdf Click here for additional data file.

# Peer review: Decision Letter 1

**DOI:** 10.1371/journal.pcbi.1011646.r003

**Authors:** Pedro Mendes, Roeland M.H. Merks

23 Oct 2023

Dear Dr. Farcot,

Thank you very much for submitting your manuscript "Fluctuations in auxin levels depend upon synchronicity of cell divisions in a one-dimensional model of auxin transport" for consideration at PLOS Computational Biology. As with all papers reviewed by the journal, your manuscript was reviewed by members of the editorial board and by several independent reviewers. The reviewers appreciated the attention to an important topic. Based on the reviews, we are likely to accept this manuscript for publication, providing that you modify the manuscript according to the review recommendations.

(The reviewers only have a few last suggestions that you may want to incorporate in your manuscript. Then it can be accepted.)

Please prepare and submit your revised manuscript within 30 days. If you anticipate any delay, please let us know the expected resubmission date by replying to this email.

When you are ready to resubmit, please upload the following:

[1] A letter containing a detailed list of your responses to all review comments, and a description of the changes you have made in the manuscript. Please note while forming your response, if your article is accepted, you may have the opportunity to make the peer review history publicly available. The record will include editor decision letters (with reviews) and your responses to reviewer comments. If eligible, we will contact you to opt in or out

[2] Two versions of the revised manuscript: one with either highlights or tracked changes denoting where the text has been changed; the other a clean version (uploaded as the manuscript file).

Important additional instructions are given below your reviewer comments.

Thank you again for your submission to our journal. We hope that our editorial process has been constructive so far, and we welcome your feedback at any time. Please don't hesitate to contact us if you have any questions or comments.

Sincerely,

Roeland M.H. Merks, Ph.D

Academic Editor

PLOS Computational Biology

Pedro Mendes

Section Editor

PLOS Computational Biology

***********************

A link appears below if there are any accompanying review attachments. If you believe any reviews to be missing, please contact ploscompbiol@plos.org immediately:

Reviewer's Responses to Questions

**Comments to the Authors:**

**Please note here if the review is uploaded as an attachment.**

Reviewer #1: All of my major concerns regarding the previous version have been sufficiently addressed.

I only have a few minor comments:

I appreciate the inclusion of Fig. 6 in the materials and methods section. It might also be worthwhile to mention the figure in the results section, where the model is introduced. Maybe something like “See Materials and methods for equations and overview figure” in line 129.

ll. 179 “This lower level of multistability is consistent with experimental observations, which do not typically report high numbers of auxin response maxima.” -> reference(s)?

l. 247 “The Case 2” -> “The case 2 […]”

ll. 253 This sentence is rather difficult to read: “One observation that can be made is that for intermediate values of T, near the tip of the domain auxin concentration oscillates.”

Maybe: “One observation that can be made is that the auxin concentration oscillates near the tip of the domain for intermediate values of T.”

Or at least a second comma: “One observation that can be made is that for intermediate values of T, near the tip of the domain, auxin concentration oscillates.”

Reviewer #2: The authors have done an excellent job in polishing their manuscript, and writing a fascinating story combining dynamical systems modelling and important biological insights. Below are some minor comments that they may consider, but I have no reservations for recommending this manuscript to be published. Well done!

Line 175: The notation "(10X)" could be spelled out to be more precise as "(10 times)".

Line 557: A space is missing between the . and the word "Plants".

Line 579: "Fewer" would be better than "less".

Reviewer #3: The authors have taken my comments into account and I am satisfied with their answers and with the textual changes in the new version of their manuscript.

**********

**Have the authors made all data and (if applicable) computational code underlying the findings in their manuscript fully available?**

The PLOS Data policy requires authors to make all data and code underlying the findings described in their manuscript fully available without restriction, with rare exception (please refer to the Data Availability Statement in the manuscript PDF file). The data and code should be provided as part of the manuscript or its supporting information, or deposited to a public repository. For example, in addition to summary statistics, the data points behind means, medians and variance measures should be available. If there are restrictions on publicly sharing data or code —e.g. participant privacy or use of data from a third party—those must be specified.

Reviewer #1: Yes

Reviewer #2: Yes

Reviewer #3: Yes

**********

PLOS authors have the option to publish the peer review history of their article (what does this mean?). If published, this will include your full peer review and any attached files.

If you choose “no”, your identity will remain anonymous but your review may still be made public.

**Do you want your identity to be public for this peer review?** For information about this choice, including consent withdrawal, please see our Privacy Policy.

Reviewer #1: No

Reviewer #2: **Yes: **Andrew L. Krause

Reviewer #3: No

Figure Files:

While revising your submission, please upload your figure files to the Preflight Analysis and Conversion Engine (PACE) digital diagnostic tool, https://pacev2.apexcovantage.com. PACE helps ensure that figures meet PLOS requirements. To use PACE, you must first register as a user. Then, login and navigate to the UPLOAD tab, where you will find detailed instructions on how to use the tool. If you encounter any issues or have any questions when using PACE, please email us at figures@plos.org.

Data Requirements:

Please note that, as a condition of publication, PLOS' data policy requires that you make available all data used to draw the conclusions outlined in your manuscript. Data must be deposited in an appropriate repository, included within the body of the manuscript, or uploaded as supporting information. This includes all numerical values that were used to generate graphs, histograms etc.. For an example in PLOS Biology see here: http://www.plosbiology.org/article/info%3Adoi%2F10.1371%2Fjournal.pbio.1001908#s5.

Reproducibility:

To enhance the reproducibility of your results, we recommend that you deposit your laboratory protocols in protocols.io, where a protocol can be assigned its own identifier (DOI) such that it can be cited independently in the future. Additionally, PLOS ONE offers an option to publish peer-reviewed clinical study protocols. Read more information on sharing protocols at https://plos.org/protocols?utm_medium=editorial-email&utm_source=authorletters&utm_campaign=protocols

References:

Review your reference list to ensure that it is complete and correct. If you have cited papers that have been retracted, please include the rationale for doing so in the manuscript text, or remove these references and replace them with relevant current references. Any changes to the reference list should be mentioned in the rebuttal letter that accompanies your revised manuscript.

*If you need to cite a retracted article, indicate the article’s retracted status in the References list and also include a citation and full reference for the retraction notice.*

# Author comment: Author response to Decision Letter 1

**DOI:** 10.1371/journal.pcbi.1011646.r004

31 Oct 2023

Attachment Submitted filename: ResponseLetter2.pdf Click here for additional data file.

# Editor report: Decision Letter 2

**DOI:** 10.1371/journal.pcbi.1011646.r005

**Authors:** Pedro Mendes, Roeland M.H. Merks

1 Nov 2023

Dear Dr. Farcot,

We are pleased to inform you that your manuscript 'Fluctuations in auxin levels depend upon synchronicity of cell divisions in a one-dimensional model of auxin transport' has been provisionally accepted for publication in PLOS Computational Biology.

Before your manuscript can be formally accepted you will need to complete some formatting changes, which you will receive in a follow up email. A member of our team will be in touch with a set of requests.

Please note that your manuscript will not be scheduled for publication until you have made the required changes, so a swift response is appreciated.

IMPORTANT: The editorial review process is now complete. PLOS will only permit corrections to spelling, formatting or significant scientific errors from this point onwards. Requests for major changes, or any which affect the scientific understanding of your work, will cause delays to the publication date of your manuscript.

Should you, your institution's press office or the journal office choose to press release your paper, you will automatically be opted out of early publication. We ask that you notify us now if you or your institution is planning to press release the article. All press must be co-ordinated with PLOS.

Thank you again for supporting Open Access publishing; we are looking forward to publishing your work in PLOS Computational Biology. 

Best regards,

Roeland M.H. Merks, Ph.D

Academic Editor

PLOS Computational Biology

Pedro Mendes

Section Editor

PLOS Computational Biology

***********************************************************

# Editor report: Acceptance letter

**DOI:** 10.1371/journal.pcbi.1011646.r006

**Authors:** Pedro Mendes, Roeland M.H. Merks

10 Nov 2023

PCOMPBIOL-D-23-00794R2 

Fluctuations in auxin levels depend upon synchronicity of cell divisions in a one-dimensional model of auxin transport

Dear Dr Farcot,

I am pleased to inform you that your manuscript has been formally accepted for publication in PLOS Computational Biology. Your manuscript is now with our production department and you will be notified of the publication date in due course.

The corresponding author will soon be receiving a typeset proof for review, to ensure errors have not been introduced during production. Please review the PDF proof of your manuscript carefully, as this is the last chance to correct any errors. Please note that major changes, or those which affect the scientific understanding of the work, will likely cause delays to the publication date of your manuscript. 

Soon after your final files are uploaded, unless you have opted out, the early version of your manuscript will be published online. The date of the early version will be your article's publication date. The final article will be published to the same URL, and all versions of the paper will be accessible to readers.

Thank you again for supporting PLOS Computational Biology and open-access publishing. We are looking forward to publishing your work! 

With kind regards,

Zsofi Zombor

PLOS Computational Biology | Carlyle House, Carlyle Road, Cambridge CB4 3DN | United Kingdom ploscompbiol@plos.org | Phone +44 (0) 1223-442824 | ploscompbiol.org | @PLOSCompBiol
